# Medical Schools

**Published:** 1845-10-01

**Authors:** 


*Medical Schools.*—The season is now at hand when the medical student is to decide at what institution he will seek instruction for the coming winter. As it is customary for medical institutions to advertise their advantages, terms, &c. through medical and other journals, and by means of circulars which are extensively distributed, students are enabled readily to obtain what general information they require, before determining to which to give their preference. As regards the relative merits of different schools, we have not data sufficient to qualify us to speak ; nor have we any disposition to make invidious distinctions in order to influence the minds of those students and preceptors who may do us the honor to read our remarks. We accord to all the merit of being honestly desirous of fulfilling their obligations to the pupil and the profession, and we sincerely believe that the majority of those who are invested with the responsibilities of teaching the various departments of medical science, are competent to the duties involved. That there are evils and abuses in some of our medical schools which require correction, all who have bestowed any observation and reflections on the subject, must admit. Of these, in our capacity of journalists, we shall not hesitate to speak as occasions offer, without intending, at any time, to apply our remarks to any particular institution.

Medical schools have increased in number of late years, so that there can hardly be said, at the present time, to be any deficiency. We do not, however, regard the fact that they are numerous, as necessarily leading to bad consequences. So far as an honorable spirit of emulation is developed and promoted by competition, it may be expected that the means of education will improve, rather than diminish, in proportion to the number of schools. But if instead of this commendable spirit, they vie with each other in offering to the pupil unworthy inducements, and in resorting to intriguing, dishonorable methods to increase the number of their students, their influence upon medical education and the profession is of the most pernicious character. If it be tacitly understood, for example, that at certain schools the acquirements of the pupil are but slightly scrutinised in admitting him to graduation, the public, the profession, and the pupil himself, alike, receive a severe injury, in so far as this consideration exerts its intended influence. It is needless to say that this course is reprehensible in the extreme. So, also, deception in any form, underbidding in price. and any of the various methods of electioneering and puffing, obviously tend to degrade the profession, and institutions known to adopt such means of advancing their interests, should be scouted by all who have the honor and usefulness of the profession at heart. We trust and believe that it would not be an easy matter to cite many, if any instances in which these enormities are practiced. There is, however, one custom more or less prevalent, which, although we would not include it in the same category in a moral point of view, is (as it seems to us) hardly less injurious in its consequences. We allude to the custom of receiving students at medical schools upon credit. Many of our schools offer this inducement to a greater or less extent. This custom in various ways exerts a detrimental influence. It does injustice to the teacher, since it is not to be supposed that he can afford to bestow his time and labor in this, more than in other departments of effort, without an adequate compensation ; and hence, it follows as a legitimate result, that institutions who adopt this custom are unable to command the same ability in their teachers as those who pursue a different system. All the facilities of instruction, also, dependant on the pecuniary resources of the institution, (and the fees from pupils generally constitute the chief resources of medical schools) will be impaired by a system, the effect of which is, that a large portion of the class receive instruction gratuitously. No institution can pursue this course and do proper justice to its pupils, and to the cause of medical education. It involves a practical discrimination which is an imposition upon those who do not ask for credit, and, in reality, obliges them to support the school for the benefit of those who contribute nothing toward it.

Its tendency is to cheapen the value of a medical education in public estimation, since it is an universal habit to estimate the real worth of any thing by what it costs. But more than all, it exerts a direct influence to lower the standard of medical attainment, and depreciate the honor and usefulness of the profession, by operating like a bounty for young men to engage in the study of medicine who are in no wise qualified either by preliminary education, habits of application, or elevated views. It is due to this evil, in a great measure, that so many enter the profession whose connection with it results in disappointment and mortification to themselves, and is a reproach to the profession.

If, then, we were to advise the medical student about to attend lectures, we should say, go prepared to pay the fees required, and give preference to an institution where this is expected of all. Let physicians generally, give this advice to their pupils, and the evil of which we have spoken will be speedily corrected.

There are other matters connected with the subject of medical schools, upon which we have some remarks to offer; but these we must defer for a future number. The subject is one of great importance, and we mean to discuss it freely and independently.

In the preceding remarks, we have considered *credit* as equivalent to a gratuitous attendance upon lectures. That it is so, is, of course, not true in a rigid sense, since a proportion of those who obtain credit ultimately fulfil their obligations to pay. If, however, we have been correctly informed by those acquainted with the practical working of the system, this proportion is not large. For those who are able to give satisfactory security for payment, the system is quite superfluous, since these persons ought to be able to obtain the facilities they require, from the circle of their immediate friends, or from those whose business is to lend money upon satisfactory security. They ought not to ask their teachers to sustain toward them the additional relation of Bankers; nor would they, if the system did not exist. But, being customary, they conclude they may as well avail themselves of it. Institutions, hence, deprive themselves of much of the immediate avails of teaching which they might otherwise have, and in this way inflict upon themselves a pecuniary injury. We would sympathize, as fully as any, with the situation of the pupil earnestly desirous of entering the profession, but repelled by impoverished means; yet, regarding the condition of the profession as affected by removing this obstacle in all cases, it would be far better for the pupil himself to submit to the necessary exertion, deprivations, and delay, than for the present state of things to continue, although it may seem to contribute to his immediate advantage.

